# Requirement for expert histopathological assessment of ovarian cancer and borderline tumours

**DOI:** 10.1054/bjoc.1999.0994

**Published:** 2000-02

**Authors:** P S Sengupta, J H Shanks, C H Buckley, W D J Ryder, J Davies, K Reynolds, R J Slade, H C Kitchener, G C Jayson

**Affiliations:** Cancer Research Campaign, Department of Medical Oncology,Histopathology,Medical Statistics,Gynaecology Oncology, Christie Hospital National Health Trust, Withington, Manchester, M20 4BX, UK; Department of Reproductive Pathology, St Mary's Hospital for Women and Children, Whitworth Park, Manchester, M13 0JH, UK

**Keywords:** ovarian tumour, borderline, pathology, survival

## Abstract

The distinction between borderline ovarian tumours (BOT) and ovarian carcinoma is made by histopathological assessment. Of 64 patients managed according to institutional BOT protocols, 27 (42%) had been referred with a diagnosis of ovarian carcinoma that was subsequently changed to BOT following histopathological review. The 70% 6-year event-free survival of the patients with a revised diagnosis was not significantly different from those who were referred with a diagnosis of BOT. This change in diagnosis is important as it avoids the need for chemotherapy for most patients and results in patients receiving appropriate information concerning prognosis. Interestingly, 24 patients (38.1%) reported a family history of epithelial cancer, a finding that has not been reported previously.© 2000 Cancer Research Campaign


British Journal of Cancer (2000) 82(4), 760–762
© 2000 Cancer Research Campaign
DOI: 10.1054/ bjoc.1999.0994, available online at http://www.idealibrary.com on IDEAL®

Short Communication

### Requirement for expert histopathological assessment of ovarian cancer and borderline tumours

**PS Sengupta[1], JH Shanks[2], CH Buckley[5], WDJ Ryder[3], J Davies[1], K Reynolds[4], RJ Slade[4], HC Kitchener[4] and GC Jayson[1]**

[1]Cancer Research Campaign, Department of Medical Oncology, Departments of [2]Histopathology, [3]Medical Statistics and [4]Gynaecology Oncology, Christie Hospital National Health Trust, Withington, Manchester, M20 4BX, UK; [5]Department of Reproductive Pathology, St Mary's Hospital for Women and Children, Whitworth Park, Manchester, M13 0JH, UK

**Summary** The distinction between borderline ovarian tumours (BOT) and ovarian carcinoma is made by histopathological assessment. Of 64 patients managed according to institutional BOT protocols, 27 (42%) had been referred with a diagnosis of ovarian carcinoma that was subsequently changed to BOT following histopathological review. The 70% 6-year event-free survival of the patients with a revised diagnosis was not significantly different from those who were referred with a diagnosis of BOT. This change in diagnosis is important as it avoids the need for chemotherapy for most patients and results in patients receiving appropriate information concerning prognosis. Interestingly, 24 patients (38.1%) reported a family history of epithelial cancer, a finding that has not been reported previously. © 2000 Cancer Research Campaign

**Keywords:** ovarian tumour; borderline; pathology; survival

Borderline epithelial ovarian tumours (BOT) were described 70 years ago as semimalignant tumours (Taylor, 1929), although diagnostic criteria were not refined until the early 1970s (International Federation of Gynaecology and Obstetrics, 1971; Serov et al, 1973). The diagnosis is made on histological criteria that there is epithelial proliferation, multilayering and atypical cytological features but an absence of destructive stromal invasion (Scully et al, 1979). The majority of these tumours are of serous or mucinous subtype, the latter being of intestinal or Müllerian type.

BOT can also be classified according to the presence of extra-ovarian, peritoneal disease. While peritoneal disease (implants) may be seedlings (superficial metastases) in the pelvic peritoneum, many of them represent foci of peritoneal metaplasia. The epithelium in these latter foci may be benign (endosalpingiosis) but may show the same characteristics as that in the ovarian neoplasm (Gershenson et al, 1990; Bell et al, 1991; Kurman and Trimble, 1993; Michael et al, 1993). Serous implants may also be subdivided into non-invasive (desmoplastic and epithelial) and invasive types (Michael and Roth, 1986; Bell et al, 1988); the latter can be treated as an invasive malignant tumour (Bell et al, 1988). Furthermore serous borderline tumour with microinvasion (individual or clusters of cells) (Tavassoli et al, 1988; Bell and Scully, 1990) has also been described. Mucinous tumours of intestinal type can be associated with pseudomyxoma peritoneii and there is evidence that some of these tumours may be metastases from a primary adenoma or adenocarcinoma of the vermiform appendix (Hinson and Ambrose, 1998).

The survival of patients with BOT is good. In a review of 953 patients that excluded those with invasive implants the survival of patients with stage I disease and stage III disease was 99% and 92% respectively at a median of 7 years follow-up (Kurman and Trimble, 1993). However, other reports have shown that the disease can follow an indolently progressive course with a 30% relapse rate over 7 years after diagnosis (Gershenson et al, 1998). Nevertheless, these survival statistics are significantly better than those for ovarian cancer.

The principal modality of treatment for BOT is surgery, whereas ovarian cancer is largely treated with surgery and chemotherapy. Thus the incorrect ascription of a diagnosis of ovarian cancer to a patient who has BOT will result in the patient receiving chemotherapy and incorrect information concerning long-term survival. In addition a correct diagnosis of BOT in a younger woman would allow the preservation of fertility. This study reports the results of expert histopathological review of cases that were finally managed as having BOT.

### MATERIALS AND METHODS

The pathology of all cases of ovarian neoplasms referred to the Department of Medical Oncology (Christie Hospital National Health Trust) is reviewed by pathologists with a special interest in gynaecological oncology (Serov et al, 1973). The patients who were managed as having BOT between 1988 and 1997 were included in this study. Prospective demographic information was gathered and the FIGO stage was determined through a summation of pathological, clinical and radiological findings (FIGO, 1971).

### RESULTS

Sixty-four patients with a diagnosis of BOT, following pathology review, were identified for the study. Their demographic details are shown in Table 1. The median age was 48 years (range 23–88 years) with a 5-year survival of 79% for all stages. None of the patients had a history of another cancer. Interestingly, 24 patients

*Received 7 July 1999*
*Revised 4 October 1999*
*Accepted 6 October 1999*

*Correspondence to:* GC Jayson

**Table 1** Clinicopathological characteristics of patients

| Characteristic | Total *n* = 64 (%/64) | OVCA *n* = 27 (%/27) | BOT *n* = 37 (%/37) |
|---|---|---|---|
| Histopathology | | | |
| Mucinous | 33 (51.6) | 13 (48.1) | 20 (54.1) |
| Serous | 25 (39.0) | 11 (40.7) | 14 (37.8) |
| Mixed | 4 (6.2) | 1 (3.7) | 3 (8.1) |
| Endometrioid | 1 (1.6) | 1 (3.7) | 0 (0.0) |
| Other | 1 (1.6) | 1 (3.7) | 0 (0.0) |
| Endosalpingiosis | 6 (9.4) | 2 (7.4) | 4 (10.8) |
| Non-invasive implants | 4 (6.3) | 4 (14.8) | 0 (0.0) |
| Invasive implants | 3 (4.7) | 0 (0.0) | 3 (8.1) |
| Pseudomyxoma peritoneii | 8 (12.5) | 5 (18.5) | 3 (8.1) |
| FIGO stage | | | |
| Ia | 21 (32.8) | 3 (11.1) | 18 (48.6) |
| Ib | 4 (6.3) | 1 (3.7) | 3 (8.1) |
| Ic | 13 (20.3) | 7 (25.9) | 6 (16.2) |
| II | 7 (10.9) | 5 (18.5) | 2 (5.4) |
| III | 18 (28.1) | 10 (37.0) | 8 (21.6) |
| Unknown | 1 (1.6) | 1 (3.7) | 0 (0.0) |
| Residual disease | | | |
| Nil | 38 (59.4) | 9 (33.3) | 29 (78.4) |
| Minimal (0–2 cm) | 7 (10.9) | 5 (18.5) | 2 (5.4) |
| Bulk (> 2 cm) | 12 (18.8) | 8 (29.6) | 4 (10.8) |
| Uncertain | 7 (10.9) | 5 (18.5) | 2 (5.4) |
| Performance status (KP) | | | |
| 100% | 6 (9.3) | 0 (0.0) | 6 (16.2) |
| 90% | 26 (39.1) | 10 (37.0) | 16 (43.2) |
| 80% | 15 (23.4) | 7 (25.9) | 8 (21.6) |
| 40–70% | 3 (4.7) | 1 (3.7) | 2 (5.4) |
| Unknown | 14 (21.9) | 9 (33.3) | 5 (13.5) |
| Disease status at follow-up | | | |
| Disease free | 46 (71.9) | 16 (59.3) | 30 (81.1) |
| Stable disease | 9 (14.1) | 5 (18.5) | 4 (10.8) |
| Recurrent disease | 1 (1.6) | 1 (3.7) | 0 (0.0) |
| Dead disease | 7 (10.9) | 5 (18.5) | 2 (5.4) |
| Intercurrent death | 1 (1.6) | 0 (0.0) | 1 (2.7) |

BOT: patients originally diagnosed as having borderline ovarian tumours; OVCA: patients originally diagnosed as having invasive ovarian tumours.

(38.1%) reported a family history of epithelial cancer (ovary, breast, bowel, prostate and other cancers).

Examination of the original pathology report (issued before specialist pathology review) showed that in 27 cases (42.2%) the original diagnosis was of an invasive carcinoma of the ovary. In 17 cases these were described as well differentiated tumours and in two cases as moderately differentiated tumours. The differentiation status was not described in the remaining eight cases. Twenty-three of the cases originally described as carcinomas were not associated with peritoneal involvement; the remaining four were associated with non-invasive implants. Of the cases originally described as carcinomas, 11 were FIGO stage I tumours (three stage Ia; one stage Ib; seven stage Ic), five were stage II tumours and ten stage III tumours (Table 1).

Ten of the 64 patients (including five originally described as carcinoma) with borderline tumours received chemotherapy. One of the patients that received chemotherapy had stage II disease and the rest had stage III disease. Of the nine patients that could be assessed for response, there was one complete response and one partial response. Five patients had a second laparotomy after chemotherapy.

The median follow-up for these patients was 3.1 years (range 40 days to 12.1 years). There were seven disease-related deaths and one intercurrent death; 46 patients remain alive and disease-free; nine are alive with stable disease; one is alive with recurrent

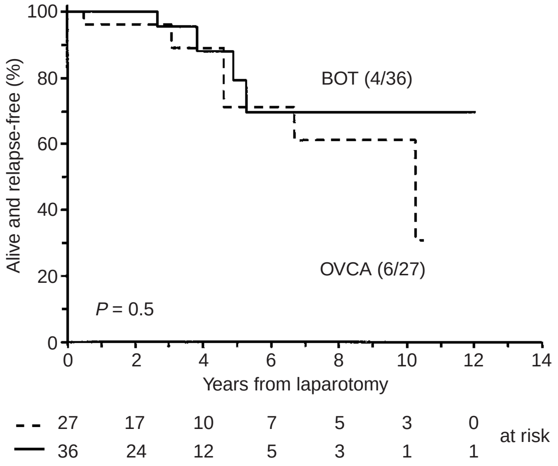

**Figure 1** Comparison of event-free survival of patients (BOT: patients originally diagnosed as having borderline ovarian tumours; OVCA: patients originally diagnosed as having invasive ovarian tumours). Note one case in the BOT group was missing necessary information and hence has been omitted here disease. Of the patients who received chemotherapy, four were disease-free at follow-up, three had stable disease, one was alive with recurrent disease, two had died of disease. In two cases there was histopathologically confirmed malignant transformation to invasive carcinoma (one death). The 5-year event-free survival (death, progression or recurrence) for stage I and II patients versus stage III was 87% and 53% respectively ($P = 0.01$). However, a comparison between the event-free survival for the group initially diagnosed with carcinomas and that of the group where the diagnosis was not changed, revealed no difference between the groups with a 70% 6-year event-free survival ($P = 0.5$) (Figure 1).

### DISCUSSION

This is a study of 64 patients with a diagnosis of borderline ovarian cancer managed at a specialist centre. The patients had been referred from other hospitals in the region and this accounts for the atypical distribution of FIGO stage in patients with BOT. Although only a minority of patients received chemotherapy, the prognosis for these tumours is good, with a 79% 5-year survival from diagnosis. However, patients with FIGO stage III disease had a worse prognosis. Interestingly, nearly 40% of the patients gave a family history of another epithelial cancer, a finding that has not been reported previously in studies of patients with BOT.

The most striking feature of this study was the finding that in 27 cases (42.2%) the original diagnosis was of invasive ovarian carcinoma prior to specialist gynaecological oncology pathology review. However, of the 27 cases where the diagnosis was revised, only four were FIGO stage Ia–Ib. The remaining cases were FIGO stage Ic–III (one unknown) and would have received chemotherapy had the diagnosis not been changed. There was no difference in event-free survival between the group that had a changed histopathological diagnosis and those with an unchanged diagnosis ($P = 0.5$). This high frequency of change in diagnosis may be due to the referral bias that led to a high proportion of advanced-stage patients in the series.

© 2000 Cancer Research Campaign

Coupled with the long event-free survival that compares with other BOT series, the implication is that borderline tumour was the correct designation. As chemotherapy is associated with early and late morbidity and mortality, most patients with BOT will be spared these toxicities and clinicians will give patients the correct information concerning prognosis. These data support the need for expert histopathological evaluation of ovarian tumours.

### ACKNOWLEDGEMENTS

We would like to thank Mrs Genny Connolly and Miss Sue Lee (data managers).

### REFERENCES

Barhill DR, Kurman RJ, Brady MF, Omura GA, Yordan E, Given FT, Kucera PR and Roman LD (1995) Preliminary analysis of the behaviour of stage I ovarian serous tumours of low malignant potential: a Gynecologic Oncology Group study. *J Clin Oncol* **13:** 2752–2756

Bell DA (1991) Ovarian surface epithelial-stromal tumours. *Hum Pathol* **22**: 750–762.

Bell DA and Scully RE (1990) Ovarian serous borderline tumours with stromal microinvasion: a report of 21 cases. *Hum Pathol* **21**: 397–403

Bell DA, Weinstock MA and Scully RE (1988) Peritoneal implants of ovarian serious borderline tumors. *Cancer* **62**: 2212–2222

FIGO (International Federation of Gynecology and Obstetrics) (1971) Classification and staging of malignant tumours in the female pelvis. *Acta Obstet Gynecol Scand* **50**: 1–7

Gershenson DM and Silva EG (1990) Serous ovarian tumors of low malignant potential with peritoneal implants. *Cancer* **65**: 578–585

Gershenson DM, Silva EG, Tortolero-Luna G, Levenback C, Morris M and Tornos C (1998) Serous borderline tumours of the ovary with non-invasive peritoneal implants. *Cancer* **83**: 2157–2163

Hinson FL and Ambrose NS (1998) Pseudomyxoma peritonei. *Br J Surg* **85**: 1332–1339

Koern J, Trope CG and Abeler VM (1993) A retrospective study of 370 borderline tumors of the ovary treated at the Norwegian Radium Hospital from 1970 to 1982. *Cancer* **71**: 1810–1820

Kurman RJ and Trimble CL (1993) The behaviour of serous tumours of low malignant potential: are they ever malignant? *Int J Gynecol Pathol* **12**: 120–127

Leake JF, Currie JL, Rosenshein NB and Woodruff JD (1992) Long-term follow-up of serous ovarian tumors of low malignant potential. *Gynecol Oncol* **47**: 150–158

Michael H and Roth LM (1986) Invasive and noninvasive implants in ovarian serous tumors of low malignant potential. *Cancer* **57**: 1240–1247

Michael H, Roth LM and Kotylo PK (1993) Recent developments in the pathology of ovarian epithelial tumours of low malignant potential and related neoplasms. *Pathol Annu* **28**: 1–22

Scully RE (1979) *Tumors of the Ovary and Maldeveloped Gonads*, AFIP Fascicle 16, 2nd Series, pp. 53–151. Armed Forces Institute of Pathology: Washington, DC

Serov SF, Scully RE and Sobin LH (1973) International histological classification and staging of tumors, No. 9. In: *Histological Typing of Ovarian Tumours*. World Health Organization: Geneva

Taylor HC Jr (1929) Malignant and semimalignant tumours of the ovary. *Surg Gynecol Obstet* **48**: 702–712

Tavassoli FA (1988) Serous tumor of low malignant potential with early stromal invasion (serous LMP with microinvasion). *Mod Pathol* **1**: 407–414

 *© 2000 Cancer Research Campaign*

